# Predictive Models of Word Reading Fluency in Hebrew

**DOI:** 10.3389/fpsyg.2018.01882

**Published:** 2018-10-09

**Authors:** Adi Shechter, Orly Lipka, Tami Katzir

**Affiliations:** ^1^Department of Learning Disabilities, Edmond J. Safra Brain Research Center, University of Haifa, Haifa, Israel; ^2^Department of Special Education, Edmond J. Safra Brain Research Center, University of Haifa, Haifa, Israel

**Keywords:** reading fluency, rapid automatized naming, phonological awareness, morphological awareness, syntax awareness, vocabulary, Hebrew

## Abstract

This study examined a multi-componential approach to reading fluency in first and third grade Hebrew speaking children. Measures of naming speed, phonological awareness (PA), morphological awareness (MA), syntax awareness, and vocabulary were administered to first (*N* = 68) and third (*N* = 67) graders. Hierarchical regression models revealed that in both grades, naming speed accounted for most of the variance in each model. However, while in the first grade, word reading fluency was also predicted by vocabulary, in the third grade, both PA and MA were significant additional predictors. Predictive models of word reading fluency in Hebrew and applied implications are proposed.

## Introduction

Reading fluency has typically been defined by referring to accurate reading at an appropriate rate (e.g., Meyer and Felton, 1999; Hudson et al., 2000; Torgesen et al., 2001; Silverman et al., 2013). Wolf and Katzir-Cohen (2001) initially expanded this definition to include a developmental-componential structure. According to their model, reading fluency incorporates not only speed and accuracy but is also influenced by various linguistic components such as vocabulary, morphological, and syntactic processes. Their broad approach has been criticized for not being empirically validated (Kame’enui and Simmons, 2001). In the current study, we seek to examine the model for the Hebrew orthography empirically through a refined focus on the prediction of word reading fluency by naming speed and multiple linguistic components in a cross-sectional design studying first and third grade children.

Wolf and Katzir-Cohen’s (2001) fluency definition builds on two theories: LaBerge and Samuels’s automaticity theory in reading and connectionist models of reading. LaBerge and Samuels (1974) described reading as a process which begins with a transformation of visual information into a series of processing stages involving visual, phonological, and episodic memory systems. The initial phase of reading, when novice readers acquire and practice their decoding strategies (Hudson et al., 2005; Speece and Ritchey, 2005), demands a considerable amount of attention which is divided between multiple subskills. Only once these skills become automatic, reading fluency is achieved (Speece and Ritchey, 2005; Kuhn et al., 2010). Connectionist models of word reading (Adams, 1990; Foorman, 1994; Caramazza, 1997; Harm and Seidenberg, 2004; Seidenberg, 2005) emphasize the ongoing interaction between linguistic features (i.e., phonological, orthographic, semantic, and syntactic processes) in word activation. (Wolf and Katzir-Cohen’s (2001) definition of reading fluency interweaves both theoretical concepts and addresses the developmental nature of the processes. That is, reading fluency is a developmental-componential structure which crystallizes gradually when underlying linguistic sublexical processes and their connections become automatic. Different subskills may play different roles in different stages of reading development.

Most studies of reading fluency have focused on the roles of phonological awareness (PA) and naming speed in this construct. (e.g., Schatschneider et al., 2002; Landerl and Wimmer, 2008; Vaessen et al., 2010; Furnes and Samuelsson, 2011; Moll et al., 2014; Lipka et al., 2016; Song et al., 2016; Tibi and Kirby, 2018). We seek to expand the scope of research by focusing on additional linguistic factors that may also play a role in word reading fluency in different stages of reading development. Furthermore, although in current years, there is an expansion of the research on the nature of reading fluency in different orthographies such as European and non-alphabetic languages (e.g., in German: Landerl and Wimmer, 2008; in Hungarian, Dutch, and Portuguese: Vaessen et al., 2010; in Chinese: Xue et al., 2013), there are scarce studies of word reading fluency in Semitic orthographies.

Several studies examined the developmental nature of reading fluency in light of the effect of orthographic consistency (e.g., in Arabic: Saiegh-Haddad, 2005; in German: Landerl and Wimmer, 2008; in English and Greek: Georgiou et al., 2008; in Finnish, Greek and English: Georgiou et al., 2012; in English and Hebrew: Katzir et al., 2012). These studies demonstrated a language-specific approach to reading fluency, which takes into account the linguistic features of the examined orthography. The studies of the Hebrew orthography have focused on reading accuracy (e.g., Ben-Dror et al., 1995; Share and Levin, 1999; Share, 2017) and less on reading fluency. In addition, there are not any studies on the different components that may predict word reading fluency at different stages of reading development.

Historically, the reading research held the axiom that PA, the ability to manipulate spoken words into speech-sound segments (Elbro, 1998, 1999; Goswami, 2000), is the most critical threshold for word reading (e.g., Goswami and Bryant, 1990; Brady and Shankweiler, 1991; Goswami, 2002). This ability is perceived as a prerequisite for successful word reading acquisition (Snowling, 2000; Kovelman et al., 2011). PA underlies the child’s knowledge of grapheme–phoneme correspondence and decoding (Wagner et al., 1997; Lambrecht Smith et al., 2008), which later enables the reader to read a word fluently (Meyer and Felton, 1999; Wolf and Bowers, 1999).

The role of PA in word reading fluency has been widely examined. During kindergarten, PA has been found to be a predictor of word reading fluency in first and second grade among Greek-speaking children (Papadimitriou and Vlachos, 2014). A similar role of PA was found in Spanish among first grade children (González-Valenzuela et al., 2016). Vaessen et al. (2010) investigated first to fourth grade children in three orthographies that differ in their degree of transparency (i.e., Hungarian, Dutch, and Portuguese). The participants were asked to read lists of high-frequency words, low-frequency words, and pseudowords, as quickly and accurately as possible in thirty seconds. The results of this study pointed to significant correlations between PA and the three examined conditions across languages.

However, the relationship between PA and reading fluency appears to be inconsistent. In a cross linguistic study, Manolitsis et al. (2009) found that while in English PA was consistently related to word reading fluency both in first and second grade, in Greek a different developmental trajectory was found. That is, a mild association was found in first grade but no correlation was observed in second grade. In another study, Georgiou et al. (2008) examined the predictors of word reading fluency in first and second grade, here too by focusing on English and Greek orthographies. In this study, PA, which was entered in the regression models together with rapid automatized naming (RAN), phonological memory, orthographic processing, and age, explained word reading fluency only among English-speaking children in first grade.

In a meta-analysis conducted by Ruan et al. (2017), the authors examined the relationships between PA and word reading fluency by comparing two different orthographies: English and Chinese. Since a stronger association was found in English, their main conclusion was that the role of PA is influenced by the specific orthography. Thus, it can be assumed that PA plays a stronger role in alphabetic systems which demand an orthography-to-phonology mapping, than in orthographies characterized by orthography-to-semantics mapping and grapheme-to-phoneme mapping.

Less is known about the relationship between PA and reading fluency in Hebrew. Unlike the learning of alphabetic scripts, the Hebrew orthography is a consonantal writing system called “abjad.” Hebrew readers acquire reading in the shallow pointed system (Shany et al., 2012; Share, 2017), a system that is classified as a regular orthography (Ravid, 2005; Katzir et al., 2012). In accordance with the syllable structure of Hebrew (Cohen-Gross, 2003), the instruction unit is a CV syllable unit (i.e., consonant-vowel combinations – “tzerufim”) (Share and Levin, 1999; Share, 2017). However, during third grade, the transition to unpointed script occurs gradually and the use of the diacritics is abandoned and forgotten (Ravid, 2005; Shany et al., 2012).

In accordance with these developmental trajectories, Share (2017) suggests that in Hebrew the relationship between PA and reading depends on decoding ambiguity, described as a U-shaped curve: at the beginning of reading acquisition, decoding ambiguity is high due to the learning of consonant and vowel correspondences. By the end of first grade, when the children acquire graphic–phonemic correspondence, PA decreases. However, in second grade and beyond, when the knowledge of vowel diacritics declines, PA increases once again. Nonetheless, it should be noted that this hypothesis refers to the process of reading acquisition, without a specific focus on reading fluency.

In a longitudinal study conducted by Shatil and Share (2003) among 349 Hebrew-speaking children, the participants were tested at two points in time: at the end of kindergarten and at the end of first grade. In this study, the correlations between word recognition (calculated as Z-scores of the average of timed and untimed conditions) and PA measures ranged from modest (i.e., initial consonant match task, *r* = 0.28, *p* < 0.05; rhyme detection and production task, *r* = 0.19, *p* < 0.05; phonemic blending task, *r* = 0.28, *p* < 0.05) to insignificant correlations (i.e., initial consonant isolation and phonological word production). However, in another study, Katzir et al. (2012) reported that in fourth grade, PA added 11% to the variance in vowelized word reading fluency, after controlling for vocabulary knowledge.

Beyond the unique role of PA in reading performance, Wolf and Bowers (1999) were the first to classify naming speed as an additional core component, especially with regard to reading fluency. During the last 20 years, the connection between naming speed and reading fluency has been well documented, by using task Denckla’s (1972), the RAN task. In this task, the individual is asked to pronounce familiar visual symbols (e.g., letters, numbers, colors, or common everyday objects) as quickly as possible (Wolf and Bowers, 1999). It was suggested that this task, referred to as “a microcosm of the reading system” (Norton and Wolf, 2012, p. 448), taps basic mechanisms that tie in to reading (Manis et al., 1999), and especially to reading fluency. These subcomponents include attentional and visual processes, stored orthographic and phonological representations, access to and retrieval of information in the phonological loop, connection to semantic and conceptual networks, and response articulation (Wolf and Bowers, 1999; Wolf and Denckla, 2005; Norton and Wolf, 2012). Empirical examination of the connection between RAN and reading fluency revealed that both access to phonological representations and serial processing explained this relationship (Georgiou et al., 2013).

An extensive body of research has shown that RAN is one of the strongest predictors of reading fluency across languages (e.g., in English: Wolf, 1984; Wolf and Bowers, 1999; Lipka, 2017; in Finnish: Puolakanaho et al., 2007, 2008; in German: Landerl and Wimmer, 2008; in French, Dutch, and Hungarian: Vaessen et al., 2010; in Italian: Tobia and Marzocchi, 2014; in Spanish: González-Valenzuela et al., 2016; in Greek: Papadopoulos et al., 2016; in Chinese: Song et al., 2016; in Hebrew: Lipka et al., 2016). Specifically, the alphanumeric subtests have proven to be strong predictors of word reading fluency in English and to a larger degree in regular orthographies (Wolf et al., 1994; Wimmer et al., 2000). Yet, less is known regarding its role in early versus later stages of reading acquisition.

Wolf and Katzir-Cohen’s (2001) model emphasized the multiplicity of linguistic components that may contribute to reading fluency. The contribution of PA and naming speed cannot explain the complexity of this construct. Instead, according to the proposed fluency definition, word reading fluency is dependent on morphological, semantic, and syntactic processes that together play an active role in word reading fluency.

Morphological awareness (MA) has been defined as awareness of the morphemic structure of words and the explicit ability to produce and manipulate it (Carlisle, 1995). Knowledge of morphemes, the smallest units of meaning within a word, allows the reader to uncover the word’s meaning through its prefix, suffix, and root (Deacon and Kirby, 2004; Carlisle, 2010). Thus, MA has been found related to word reading ability and development (e.g., Brittain, 1970; Freyd and Baron, 1982; Tyler and Nagy, 1990; Snow et al., 2005). Morphological knowledge has been found to accelerate both recognition and retrieval of orthographic units of the word, which, in turn, stimulate the speed of word reading (in English: Berninger et al., 2001; in Italian: Burani et al., 2008; in Spanish: D’alessio et al., 2018).

Several studies pointed to language specific relationships between MA and reading fluency. In English, for example, Kirby et al. (2012) revealed that MA functions as a predictor of word reading fluency. Furthermore, they pointed to its developmental nature. That is, in third grade MA predicted word reading fluency better than in first and second grade, after controlling for intelligence and PA. In the study conducted by Desrochers et al. (2017), MA also predicted word reading fluency both in English and French, after controlling for PA and RAN. However, this result was not duplicated in Greek (Desrochers et al., 2017; Diamanti et al., 2017; Manolitsis et al., 2017). Thus, it appears that the role of MA is stronger in opaque orthographies characterized by ambiguous spelling–sound correspondence then in transparent orthographies, in which the letter–sound correspondence is direct.

The Hebrew morphology is characterized by a linguistic density, manifested in both derivational and inflectional word formation (Vaknin-Nusbaum et al., 2015). The derivational morphology of Hebrew has two basic features, a root and a word pattern (Deutsch et al., 2000), which usually compose a word in a nonlinear combination structure (Schiff and Rave, 2006). However, in this study, we focused on the inflectional morphology, which includes grammatical features such as tense, aspect, mood, polarity, person, number, gender, and case (Wilson et al., 2014). The Hebrew inflectional morphology contains complex, inconsistent, and irregular cases that hinder its predictability, and also cases that are uncommon in the spoken form and reflect literacy expertise (Schiff et al., 2011). Therefore, we chose to assess this morphological system with regard to reading fluency. We found a single evidence of the connection between MA and reading fluency in Hebrew. In the study, conducted by Cohen-Mimran (2009) among fifth graders, a moderate positive correlation was found between possessive nouns and text reading fluency (*r* = 0.4, *p* < 0.01). However, word level was not assessed.

According to Wolf and Katzir-Cohen (2001) approach, reading fluency involves activation of both sublexical and lexical processes and occurs through the connection between phonological representation and meaning (Adams, 1990). Vocabulary, which is used as the index of semantic knowledge (Ricketts et al., 2008), is one of the linguistic comprehension components necessary for skilled reading (Hoover and Gough, 1990). This component has been consistently linked to word reading development (Ouellette, 2006; Wise et al., 2007; Ricketts et al., 2008; Katzir et al., 2012), but less to fluency.

Walley et al. (2003) suggested that the connection between vocabulary and word reading development is mediated by the child’s phonological skills. That is, semantic knowledge converts into phonological representations which are the basis of word reading. The triangle model (Harm and Seidenberg, 1999, 2004) described word reading development by the interaction of three components: phonology, orthography, and semantics. The initial phase of word reading learning occurs through phonology-to-orthography mapping but gradually, the role of vocabulary increases. Then, word reading involves top-down processes and occurs through orthography-to-phonology mapping, via semantics (Plaut et al., 1996; Harm and Seidenberg, 2004). This triangle model supplies a theoretical explanation for exception words in English, when the reader cannot rely on direct decoding (e.g., Bowey, 2001; Nation and Snowling, 2004). Ricketts et al. (2008) argued that vocabulary contributes to each word reading, beyond its regularity or familiarity. With regard to reading fluency, Wolf et al. (2000) argued that semantic knowledge enables rapid recognition and retrieval of the printed word. Instead of letter-to-letter decoding, the more meanings a child knows for a specific word, the faster he or she will retrieve it while reading (Wolf et al., 2000; Nation and Snowling, 2004).

The connection between vocabulary and word reading in the initial phases of reading acquisition was reported in Greek, when vocabulary knowledge in kindergarten was mildly related with word reading fluency in first and second grade (*r* = 0.33, *p* < 0.001; Manolitsis et al., 2017). In another study, Muroya et al. (2017) found no association between semantic knowledge at the beginning of first grade and word reading fluency in the middle of first and second, in both Hiragana and Kanji.

The relationship between vocabulary and word reading fluency appears to be stronger among skilled readers in English. Kim et al. (2010) reported a moderate correlation (*r* = 0.52, *p* < 0.01) between these variables in a sample of 294 children in grades 4–6. In another study, Katzir et al. (2012) examined the prediction of word reading fluency among English and Hebrew speaking children in fourth grade. Whereas vocabulary predicted word reading fluency in English, it did not function as a predictor in Hebrew (both pointed and unpointed scripts) after controlling for PA. These findings might point to a stronger contribution in opaque orthographies among proficient readers. However, it appears that there is a lack of empirical knowledge concerning the predictive role of vocabulary to word reading fluency in early stages of reading acquisition in Hebrew.

Wolf and Katzir-Cohen (2001) suggested that syntax is an additional underlying factor of word reading fluency. However, since syntactic awareness refers to the ability to understand grammar rules and sentences construction (Layton et al., 1998), its relevance at the word level is questionable. Willows and Ryan (1986) provided initial evidence for the connection between syntactic awareness and word reading in first-to-third grades. Further, Tunmer et al. (1988) and Tunmer and Hoover (1992) pointed to the unique contribution of syntactic awareness in word reading development. They argued that syntactic awareness is one of the most important metalinguistic abilities involved in decoding proficiency, beyond PA. That is, during the acquisition of grapheme-to-phoneme mapping, the reader uses his syntactic awareness in order to solve decoding ambiguity of unfamiliar printed words, especially in the case of homographic spelling patterns (for example, clear-bear) and exception words.

Mokhtari and Thompson (2006) found a connection between syntax and text reading fluency. However, Klauda and Guthrie (2008) reported on a correlation of *r* = 0.62 between syntactic processing and word reading fluency among fifth graders. Rumelhart (1994) claimed that syntactic knowledge, as a top-down process of language comprehension, promotes word reading fluency. Apart from these scarce studies, there is still a dearth of literature with regard to the connection between syntax and word reading fluency.

In summary, each of these components suggested by Wolf and Katzir-Cohen (2001) has been found to relate to word reading fluency. However, the current literature emphasizes the differences between shallow and opaque orthographies as well as age differences. In addition, the concurrent contributions of these components to reading fluency have not been sufficiently studied, especially with regard to the Hebrew orthography.

A number of studies investigated reading fluency through a cross-linguistic approach (e.g., Georgiou et al., 2008; Katzir et al., 2012). Yet, there is a lack of broad and deep observations regarding reading fluency in the Hebrew orthography. Therefore, we seek to explore the nature of reading fluency by portraying its trajectories among Hebrew-speaking children.

In the initial phase of reading acquisition in Hebrew, the pointed script lets readers rely on letters and vowel signs (called nikud) for accurate reading (Share and Bar-On, 2017) and promotes rapid and early mastery (Share and Levin, 1999; Shany et al., 2012). Hence, when the reader exhibits a ceiling effect in accuracy, the role of speed and fluency increases (e.g., Breznitz and Misra, 2003; Breznitz, 2006). However, Share and Bar-On (2017) claimed that the transition to unpointed script, during third grade, inhibits the development of reading fluency.

A previous study, conducted by Katzir et al. (2012), investigated word reading fluency among fourth grade Hebrew-speaking children. By examining their reading in two Hebrew scripts, pointed and unpointed, the results demonstrated separate processes that occur during word reading fluency in each script. In our study, reading fluency will only be examined through the pointed script, before the transition to the unpointed system has been completed.

### The Current Study

The literature mentioned above reveals empirical gaps that we seek to address. To date, it appears that reading fluency has not been sufficiently investigated through a multi-componential perception. Also, to our knowledge, no study has examined the prediction of word reading fluency in the Hebrew orthography by assessing a comprehensive view of the underlying components that might play a role. We suggest a cross-sectional examination of these issues at two points in reading development: first and third grade. Accordingly, this study was conducted in order to answer the following question: what are the predictors of word reading fluency in first and third grade?

## Materials and Methods

### Participants

The sample consisted of 135 Israeli children: 68 first graders (average age = 6 years 5 months; 33 girls, 35 boys) and 67 third graders (average age = 8 years 7 months; 32 girls, 35 boys). The participants were drawn from two elementary schools located in different socioeconomic neighborhoods, one medium low (*N* = 43 first graders; *N* = 42 third graders) and one medium high (*N* = 25 first graders; *N* = 25 third graders), in northern Israel. In first grade, two participants were omitted from the original data since they were not able to read words. Two additional students were classified as outliers on each reading task and were omitted as well. In third grade, one student was omitted since he was an immigrant. All students were proficient in Hebrew, with scores in the average range in all linguistic measurements.

### Measurements

#### Reading Fluency Measurements

Word reading fluency was measured by two oral reading tasks which include a list of isolated pointed words: (1) “Aleph-Taph” (Shany et al., 2006). A horizontal list which contains 38 nouns varying in frequency, length, and morphological structure. The participants were asked to read aloud the required list as accurately and quickly as possible. Fluency score was computed by calculating correct words read per minute. Cronbach’s alpha for second grade is 0.90. (2) Test of word reading efficiency (TOWRE; Schiff et al., unpublished, adapted from Wagner et al., 2001) contains 104 words arranged in four columns and organized by increasing level of difficulty. The score was computed by calculating correct words read within 45 s. Cronbach’s alpha is 0.95. We chose to use two different tasks since the first task was taken from the only individually administered test battery with national norms available in Hebrew (“Aleph-Taph”) and the second task is a theoretically accepted tool. Both tools have high reliability. In this manner, we strengthened the statistical validity of our suggested models.

#### Rapid Automatized Naming (RAN)

Naming speed was measured by three RAN tasks: digits, letters, and objects (“Aleph-Taph,” Shany et al., 2006). Participants were instructed to name the stimuli as quickly and accurately as possible. The task contains a training phase in which the participants were required to name five symbols in an untimed condition. This task contains 50 stimuli arranged randomly in a table of five rows and 10 columns. Reported test–retest reliability across ages for this task is 0.92 (Wolf and Denckla, 2005).

#### Phonological Awareness

Phonological awareness was measured by a syllable omission task (“Aleph-Taph,” Shany et al., 2006). This task contains 14 items and the target syllables appear in different positions (i.e., initial, medial, and final). Each demanded manipulation produced another real word (e.g., “Say geshem” = rain, “Now say it without the /ge/.” Response: “shem” = name). The reported Cronbach’s α for second grade is 0.79.

#### Morphological Awareness

Inflectional morphology was tested orally by identification of possessive nouns (Vaknin-Nusbaum et al., 2015). The possessive nouns task included nine items divided equally into three (i.e., sheli = my, shelo = his, shelahem = their, e.g., “Habayit shelahem hu…” “beytam” – “The house belonging to them is…” “their house”). The participants were asked to produce a real word, in accordance with linguistic regularity and based on common vocabulary knowledge. Cronbach’s α for this task is 0.81 for second grade.

#### Vocabulary

The Wechsler Intelligence Scale for Children (WISC-III, Wechsler, 1991) was used as a measure of vocabulary. Participants were asked to define 25 heard words. The scores were 0 points for an incorrect answer, 1 point for poor definition, and 2 for good definition. The test ended after five wrong consecutive answers.

#### Syntax Awareness

The task (Shatil and Share, 2003) consisted of 20 sentences, but only six of them were grammatical and the rest had a specific error. Participants were instructed to listen to the sentence read by the examiner and then asked to decide whether it is an intact sentence. If not, they were required to produce a whole sentence, based on the false one. We chose to score this task differently than the original manual, meaning that the scores ranged from 0 to 3: 0 for incorrect judgment (e.g., the child said that the sentence “yesterday we will see a movie” is intact), 1 for right judgment but wrong production (e.g., “Tomorrow we saw a movie”), 2 for right judgment but production of a sentence that was not adequate for the relevant error (e.g., “It is possible to watch a movie only tomorrow”), and 3 for right judgment and production (e.g., “Yesterday we saw a movie”). The total score, which originally ranged from 0 to 60, was computed as an accuracy percentage. Cronbach’s α for this task is 0.83.

#### Digit Span

Working memory was controlled by using the Digit Span backward task (WISC-R; Wechsler, 1974). Participants were asked to recall digits sequences of increasing length in reverse order, presented orally by the examiner. Each length level contains two trials, ranged from two to the maximum of eight digits. If the child failed on two out of the two trials in a length level, the testing was discontinued. Total score was calculated as the number of sequences that child recall in the correct order.

### Procedure

Data were collected during June, the last month of the school year. Graduate students administered the battery, which included tasks they were trained to use. Each student was examined individually, in a quiet room within the school setting. The number of sessions for each student ranged from a single meeting to two meetings, according to the child’s concentration, his or her mood, and contemporary needs of the school environment.

## Results

**Table 1** contains the descriptive statistics for each measure, used separately for first grade and third grade, as well as one-way MANOVA results. The results demonstrate that third graders outperformed first graders in all tasks, a pattern that reinforces the existence of two distinguishable sections. Since this premise was confirmed, our further analyses were divided into two groups: first grade and third grade.

**Table 1:** Means, standard deviations (SD), and one-way MANOVA results for first grade (*N* = 68) and third grade (*N* = 67).

	First grade		Third grade			
*Measures*	*M*	*SD*	*M*	*SD*	*F*	η*_p_*^2^
Reading fluency – TOWRE (correct words per 45 s)	29.59	10.54	58.79	16.74	147.45^∗∗^	0.53
Reading fluency – “Alef Taf” (correct words per minute)	13.33	7.44	37.05	16.58	103.19^∗∗^	0.45
Syllable omission – 14 items (raw score)	11.42	2.55	12.85	1.76	12.78^∗∗^	0.09
Possessive nouns – nine items (raw score)	4.31	2.43	6.69	1.79	36.78^∗∗^	0.22
Syntax awareness – 20 items (raw score)	27.13	9.30	39.60	5.85	79.57^∗∗^	0.38
Vocabulary – 22 items (raw score)	11.41	4.57	19.15	5.39	70.96^∗∗^	0.36
RAN digits – 50 items (time)	44.88	10.79	31.36	7.88	62.61^∗∗^	0.33
RAN letters – 50 items (time)	48.82	11.62	37.54	7.60	44.00^∗∗^	0.25
RAN objects – 50 items (time)	63.59	14.75	47.84	9.00	55.50^∗∗^	0.30
Digit span – 14 items (raw score)	3.02	0.89	4.00	1.36	24.42^∗∗^	0.16

*^∗^*p* < 0.05; ^∗∗^*p* < 0.01*.

We used the same sequence of analyses in both sections: first, due to strong relationships between the two tasks of word reading fluency (*r* = 0.91 in first grade and *r* = 0.87 in third grade, *p* < 0.001), we created a word reading fluency construct for each grade. Then, we examined the correlations between the word reading fluency construct and the independent variables (i.e., RAN, PA, MA, syntax awareness, and vocabulary) in order to observe both the interrelations between the independent variables and with regard to reading fluency. Finally, we conducted a hierarchical regression analysis to reveal the amount of variance contributed by RAN, PA, MA, syntax awareness, and vocabulary to word reading fluency, after controlling for age.

### Pearson’s Correlation Analyses

With regard to word reading fluency, RAN digits was the strongest correlated variable. Also, in both grades, MA, syntax awareness, and vocabulary were mildly related to word reading fluency. However, while in first grade, a mild association was found between PA and word reading fluency, the connection between these variables was moderate in the third grade.

No correlations were found between RAN tasks and linguistic variables in the first grade. However, in third grade, RAN digits was mildly correlated with PA and vocabulary, a pattern which was not observed for the RAN letters and RAN objects. In both grades, PA was correlated with syntax awareness and vocabulary, and MA, syntax awareness, and vocabulary were moderately related to each other (**Table 2**).

**Table 2:** Correlations between word reading fluency construct, naming speed, phonological awareness, morphological awareness, syntax awareness, and vocabulary measures, in first grade (*N* = 68) and third grade (*N* = 67).

		First grade								Third grade						
		1	2		3	4	5	6	7	1	2	3	4	5	6	7
1	Word reading fluency															
2	RAN digits	-0.50^∗∗^								-0.56^∗∗^						
3	RAN letters	-0.47^∗∗^	0.70^∗∗^							-0.37^∗∗^	0.58^∗∗^					
4	RAN objects	-0.39^∗∗^	0.68^∗∗^		0.52^∗∗^					-0.37^∗∗^	0.62^∗∗^	0.53^∗∗^				
5	PA – syllable omission	0.25^∗^	-0.11		-0.17	-0.20				0.48^∗∗^	-0.37 ^∗∗^	-0.21	-0.13			
6	MA – possessive nouns	0.33^∗∗^	-0.15		-0.16	-0.17	0.14			0.30^∗^	0.05	0.04	0.01	0.12		
7	Syntax awareness	0.40^∗∗^	-0.12		-0.10	-0.02	0.25^∗^	0.47^∗∗^		0.30^∗^	-0.07	-0.15	-0.19	0.31^∗^	0.54^∗∗^	
8	Vocabulary	0.40^∗∗^	0.01		-0.00	-0.02	0.42^∗∗^	0.55^∗∗^	0.56^∗∗^	0.36^∗∗^	-0.26^∗^	-0.11	-0.13	0.32^∗∗^	0.37^∗∗^	0.44^∗∗^

*^∗^*p* < 0.05;^∗∗^*p* < 0.01*.

### Multiple Regression Analyses

Based on the correlation data, we conducted a multiple regression analysis for each grade by using the enter approach. The strongest correlated variable among the RAN tasks, RAN digits, was entered into the models as an index of naming speed. By using the same variables (i.e., RAN digits, PA, MA, syntax awareness, and vocabulary), and controlling for age, this approach allowed us to demonstrate a prediction model for word reading fluency for each section (**Table 3**).

**Table 3:** Summary of the multiple regression analysis for word reading fluency: enter approach, in first grade (*N* = 68) and third grade (*N* = 67).

		Model 1			Model 2		
	Variable	*B*	*SE*	Standardized β	*B*	*SE*	Standardized β
First grade	Reading fluency						
	Age	-0.02	0.12	-0.02	0.10	0.10	0.10
	RAN				-0.02	0.01	-0.50^∗∗^
	Vocabulary				0.03	0.02	0.29^∗^
	Phonological awareness				0.00	0.00	0.06
	Morphological awareness				0.01	0.03	0.07
	Syntax awareness				0.01	0.01	0.10
	F change	0.04			7.79
	*R*^2^	0.00			0.41
Third grade	Reading fluency						
	Age	-0.21	0.20	-0.13	-0.08	0.16	-0.05
	RAN				-0.05	0.01	-0.48^∗∗^
	Vocabulary				0.01	0.02	0.04
	Phonological awareness				0.02	0.01	0.23^∗^
	Morphological awareness				0.12	0.05	0.24^∗^
	Syntax awareness				0.01	0.02	0.07
	F change	1.07			11.09
	*R*^2^	0.02			0.49

*^∗^*p* < 0.05; ^∗∗^*p* < 0.01*.

In first grade, word reading fluency was predicted by RAN and vocabulary, after controlling for age. Taken together, the model accounted for 41% (adjusted *R*^2^ = 0.35) of the variance in word reading fluency, with effect size *f*^2^ = 0.54. In third grade, word reading fluency was predicted by RAN, PA, and MA, after controlling for age, a model that accounted for 49% (adjusted *R*^2^ = 0.44) of the variance, with effect size *f*^2^ = 0.78. The reported models remained without any significant change when we controlled for working memory.

## Discussion

This study sheds new light on reading fluency, by adding two main contributions to the literature. First, our findings support a nuanced componential and developmental model of word reading fluency. Thus, while naming speed is a powerful and consistent predictor both in first and third grade, we found different patterns concerning the interplay between linguistic components, depending on the phase of reading development. That is, in first grade, vocabulary has a significant role in word reading fluency prediction while in third grade, PA and MA add a significant contribution to the model. Second, to the best of our knowledge, this study is the first to examine word reading fluency in the Hebrew orthography by taking into account varied underlying components and their predictive role at two points in time during reading development.

### Predictive Models of Reading Fluency

Almost two decades ago, Wolf and Katzir-Cohen (2001) suggested a wide definition of reading fluency. Their definition initially emphasized the multiplicity of distinct components involved in the process of fluent reading, including both linguistic and rapid naming factors. In the following sections, we will discuss similar and different patterns that appeared in first grade and third grade, concerning each predictor observed.

Rapid automatized naming explained the most variance in each model. This finding was reported in varied orthographies (for example, Georgiou et al., 2016) and reinforces the claim that RAN is a strong predictor of word reading performance (Wolf et al., 1994). However, unexpectedly, the interactions between naming speed tasks and varied linguistic components pointed to interesting findings concerning the developmental nature of these tasks. At the end of first grade, naming speed measurements acted in the same manner: they were not associated with any linguistic component. This pattern suggests that at the initial phases of reading acquisition, naming speed is a distinct variable which does not overlap with other linguistic components. It also raises the possibility that naming speed reflects a refined cognitive ability. However, our findings revealed that after 3 years of formal instruction, naming speed tasks demonstrated different patterns. Whereas RAN letters and RAN objects maintained their specificity, RAN digits was mildly associated with vocabulary and moderately correlated with PA. Thus, RAN digits at the end of third grade was the only case which manifested the assumed connections between naming speed and phonological and semantic processes (Wolf and Bowers, 1999; Wolf and Denckla, 2005).

Vocabulary was found to be a predictor of word reading fluency at the end of first grade, but not in third grade. A possible interpretation is that after first year of schooling, Hebrew speaking readers rely on their own semantic knowledge in order to read a word fluently. Both the triangle model (Harm and Seidenberg, 1999, 2004) and the dual-route model of reading (Coltheart et al., 2001) describe the learning processes of word reading by the interactions between phonology, orthography, and semantics. According to both models, word reading can occur via the phonological pathway or via the semantic route. Our results suggest a possible interplay between PA and vocabulary in different stages in word reading fluency development. That is, as the role of PA increases, the role of vocabulary decreases.

Hebrew speaking children acquire reading using the pointed script. This shallow orthography is characterized by low ambiguity and enables rapid mastery (Share and Levin, 1999; Shany et al., 2012). According to Share’s (2017) hypothesis, at the end of first grade, the role of PA decreases, and instead, as we suggest, the role of vocabulary increases. Whereas computational models of reading (Coltheart et al., 2001; Harm and Seidenberg, 2004) were conceptualized based on the English irregularity, we focused on word reading fluency in a regular orthography. According to the observed results, we suggest that when the reader achieved decoding proficiency, vocabulary, as a top-down process, enhances the speed in which the deciphered orthographic string is recognized and retrieved (Wolf et al., 2000).

In a previous study, conducted by Manolitsis et al. (2017), a positive relationship between vocabulary in kindergarten and word reading fluency in first and second grade among Greek speaking children was reported. Our study provides further evidence that semantic knowledge, and not syntax (Tunmer et al., 1988; Tunmer and Hoover, 1992), is the linguistic comprehension component which uniquely contributes to novice reader for reading a word fluently. In light of this assumption, it might also be assumed that at this stage, general linguistic factors, such as verbal processing, underlies the observed relationship between vocabulary and word reading fluency. Whereas word reading fluency requires rapid retrieval of the printed word, oral vocabulary is about a lexical retrieval of a pronounced word. However, both actions required efficient retrieval of verbal processing at the word level (Perfetti and Lesgold, 1979).

The absence of vocabulary in the predictive model of third grade supports previous finding among Hebrew speaking children in fourth grade (Katzir et al., 2012). This pattern should be explained by focusing on the developmental trajectory which occurs at this phase: the reader gradually abandons the use of diacritics and forgets their function (Ravid, 2005; Shany et al., 2012; Share and Bar-On, 2017). Thus, it can be assumed that during the transition to the unpointed script, the symptoms of this phase are manifested in their reading, even when reading a pointed script. Specifically, at the end of third grade, the reader has to struggle with other version of the Hebrew script which supplies less phonological information. Therefore, this stage requires renewed proficiency in the phonology-to-orthography mapping, a process that might reduce the role of vocabulary.

The fact that vocabulary did not predict word reading fluency in Hebrew but seems to be a predictor in English among skilled readers (Katzir et al., 2012), might be explained by the differences between the two orthographies. That is, while Hebrew speaking third graders may struggle with a new challenge (that is, the transition to the unpointed script), the English readers are familiar with the indirect conversion between grapheme-to-phoneme (e.g., Bowey, 2001; Nation and Snowling, 2004; Ricketts et al., 2008). Thus, it appears that the predictive role of vocabulary depends both on orthography consistency and age. Specifically, according to our results, vocabulary has a stronger role than other linguistic components in two cases: in the early phases of reading acquisition in shallow orthographies and among skilled readers in ambiguous scripts.

Phonological awareness and MA are additional differences between the two grades in the prediction of word reading fluency since, according to our model, PA and MA predicted word reading fluency only in third grade. The absence of PA in first grade supplies further support for previous evidence in Hebrew (Shatil and Share, 2003) and Greek (Georgiou et al., 2008). However, MA was found to be a predictor in third grade, although we concluded that it has a stronger role in opaque orthographies than in transparent orthographies (Kirby et al., 2012; Desrochers et al., 2017; Diamanti et al., 2017; Manolitsis et al., 2017).

According to our interpretation, when knowledge of vowel diacritics decreases and decoding ambiguity increases, third graders rely on the smallest linguistic components (i.e., phonemes and morphemes), rather than the entire word (i.e., semantics), in order to read a word fluently. This pattern reinforces the validity of Share’s (2017) hypothesis concerning the relationship between PA and word reading in Hebrew. Concurrently, the predictive role of MA in word reading fluency reinforces Wolf and Katzir-Cohen (2001) approach, which was based on Adams (1990) and Berninger et al. (2001) findings that morphological processes are related to rapid recognition and retrieval of orthographic units of the word.

## Conclusion

The fact that word reading fluency is predicted consistently by specific linguistic components is productive grounds for intervention programs. These programs should take place during preschool years in preparation for fluent reading. However, explicit instruction of linguistic components is also relevant during the initial years of schooling, as a promoting element of reading fluency among skilled readers.

This study has several limitations. First, the fact that it is a cross-sectional study with a relatively small sample size for each grade might limit generalization of our results to the general population. Second, we suggest a predictive model of reading fluency in Hebrew. Thus, our study is language specific and its validity for other languages should be examined. Third, our heterogeneous sample included participants whose native language was not Hebrew (e.g., Russian and Amharic), a fact that might be influential when linguistic components are assessed. Fourth, in this study, we did not examine orthographic knowledge, which is an additional linguistic aspect of words. Also, this study did not take into account the possible contribution of non-verbal IQ as well as varied cognitive components (e.g., short-term memory and executive functions) to word reading fluency.

Further research is needed in order to address the limitations of the current study: our proposed model should be examined across languages, since each language has its own linguistic features. In addition, future research should include only native speakers. Another study could examine the proposed model by comparing L1 and L2 speakers. Also, orthographic knowledge should be observed as part of the linguistic measures. In addition, in order to be able to generalize our findings with regard to MA, a future study should expand different aspects of this component. A further study should examine the developmental-componential structure of reading fluency by taking into consideration the relative contributions of varied cognitive components. Finally, there is a need to investigate text reading fluency in the Hebrew orthography.

## Ethics Statement

This study was carried out in accordance with the recommendations of the Ethics committee of the University of Haifa, and the Ethics committee of the Ministry of Education in Israel, with written informed consent from all subjects. All subjects gave written informed consent in accordance with the Declaration of Helsinki.

## Author Contributions

AS, OL, and TK conceptualized this study. AS contributed to data collection and statistical analysis. AS and TK contributed to the writing and interpretation of the data. All authors agreed to be accountable for the content of the work.

## Conflict of Interest Statement

The authors declare that the research was conducted in the absence of any commercial or financial relationships that could be construed as a potential conflict of interest.
